# Development and validity evidence for the resident-led large group teaching assessment instrument in the United States: a methodological study

**DOI:** 10.3352/jeehp.2024.21.3

**Published:** 2024-02-23

**Authors:** Ariel Shana Frey-Vogel, Kristina Dzara, Kimberly Anne Gifford, Yoon Soo Park, Justin Berk, Allison Heinly, Darcy Wolcott, Daniel Adam Hall, Shannon Elliott Scott-Vernaglia, Katherine Anne Sparger, Erica Ye-pyng Chung

**Affiliations:** 1 Department of Pediatrics, Harvard Medical School and Massachusetts General Hospital, Boston, MA, USA; 2 Center for Educator Development, Advancement, and Research and Department of Family and Community Medicine, Saint Louis University School of Medicine, Saint Louis, MO, USA; 3 Department of Pediatrics, Cleveland Clinic Lerner College of Medicine at Case Western University, Cleveland, OH, USA; 4 Department of Medical Education, University of Illinois College of Medicine, Chicago, IL, USA; 5 Department of Pediatrics and Internal Medicine, The Warren Alpert Medical School of Brown University and Hasbro Children’s Hospital, Providence, RI, USA; 6 Department of Pediatrics, The Warren Alpert Medical School of Brown University and Hasbro Children’s Hospital, Providence, RI, USA; 7 Department of Pediatrics, Geisel School of Medicine at Dartmouth and Dartmouth-Hitchcock Medical Center, Lebanon, NH, USA; Hallym University, Korea

**Keywords:** Educational measurement, Faculty, Internship and residency, Reproducibility of results, United States

## Abstract

**Purpose:**

Despite educational mandates to assess resident teaching competence, limited instruments with validity evidence exist for this purpose. Existing instruments do not allow faculty to assess resident-led teaching in a large group format or whether teaching was interactive. This study gathers validity evidence on the use of the Resident-led Large Group Teaching Assessment Instrument (Relate), an instrument used by faculty to assess resident teaching competency. Relate comprises 23 behaviors divided into 6 elements: learning environment, goals and objectives, content of talk, promotion of understanding and retention, session management, and closure.

**Methods:**

Messick’s unified validity framework was used for this study. Investigators used video recordings of resident-led teaching from 3 pediatric residency programs to develop Relate and a rater guidebook. Faculty were trained on instrument use through frame-of-reference training. Resident teaching at all sites was video-recorded during 2018–2019. Two trained faculty raters assessed each video. Descriptive statistics on performance were obtained. Validity evidence sources include: rater training effect (response process), reliability and variability (internal structure), and impact on Milestones assessment (relations to other variables).

**Results:**

Forty-eight videos, from 16 residents, were analyzed. Rater training improved inter-rater reliability from 0.04 to 0.64. The Φ-coefficient reliability was 0.50. There was a significant correlation between overall Relate performance and the pediatric teaching Milestone (r=0.34, P=0.019).

**Conclusion:**

Relate provides validity evidence with sufficient reliability to measure resident-led large-group teaching competence.

## Graphical abstract

[Fig f2-jeehp-21-03]

## Introduction

### Background/rationale

Most countries utilizing competency-based medical education require training and assessment of residents as teachers [1,2]. An integral part of resident teaching is in large group settings of 10 or more learners, especially case conferences. In these large group settings, different instructional techniques are needed to created interactive teaching experiences as compared to more intimate small group teaching. Approximately 92% of pediatric resident-as-teacher (RAT) curricula include case-based teaching and 83% include large group teaching [3].

A review of published RAT curricula identified 11 studies for which an observer assessed resident teaching, 4 of which provided validity evidence for their assessment instruments [4]. However, none included large-group teaching, standardized training for instrument use, natural classroom settings, or emphasis on interactive teaching [5,6].

Gathering validity evidence for a robust assessment of resident-led large group teaching addresses a critical gap. Thus, we undertook a rigorous instrument development process, including a literature review, resident focus groups, and a modified Delphi panel of international experts on RAT curricula and medical education assessment [7]. We identified 6 elements of resident teaching for assessment: learning environment, goals and objectives, promotion of understanding and retention, session management, and closure. Within these elements, 11 sub-elements and 23 behaviors were defined. We conducted a pilot study of the Resident-led Large Group Teaching Assessment Instrument (Relate) on case-based large group teaching. In our pilot study, the instrument had excellent internal consistency (Cronbach’s α=0.84), but varying interrater reliability for its sub-elements [7]. Internal consistency, as measured by Cronbach’s α, measures how closely related a set of items are as a group and can be interpreted to mean that the items were all measuring the same concept, resident teaching competency.

### Objectives

We aimed to improve the instrument’s interrater reliability through assessor training and to gather sources of validity evidence through a multisite study. We examined sources of validity evidence using Messick’s unified validity framework to determine whether scores from Relate could be used to provide formative and summative feedback to residents on their large-group teaching competency. With sufficient validity evidence, Relate could be used by residents to improve their teaching, program directors to assess resident competence, and residency programs to evaluate RAT curricular efficacy.

## Methods

### Ethics statement

This study was approved by Institutional Review Boards at Mass General Brigham (protocol# 2018P000287), Dartmouth College (protocol# 00030946), and Rhode Island Hospital (protocol# 1199767). Informed consent was obtained from all subjects.

### Study design

This was a cross-sectional study for establishing validity evidence of an assessment instrument.

### Setting

This study took place at Mass General for Children, Hasbro Children’s Hospital (Brown), and Dartmouth-Hitchcock Medical Center (Dartmouth), all in the northeastern United States. Recruitment occurred in 2018 and data were collected 2018–2019.

All final year residents in the programs participate in a site-specific mandatory RAT curriculum in which they lead 2–8 case-based teaching conferences per year for a group of 10 or more learners including medical students, residents, and faculty. The requirements for the teaching conferences vary by site and residents receive varying degrees of mentorship, observation, and feedback.

### Participants

The participants were recruited from post-graduate year (PGY) 3 residents at Mass General for Children, Brown, and Dartmouth and Mass General for Children PGY4 medicine-pediatric residents in 2018. No information on gender or age was collected. Residents who opted out of study participation were excluded from the study.

### Variables

The outcome variables included rater assessment of resident performance on the 23 items of the Relate (Supplement 1) and the teaching occasion (the order of the teaching recordings for a given resident).

### Data sources/measurement

All residents who agreed to participate in the study had their teaching conferences video recorded. Individual residents had 1–7 of their teaching sessions assessed with a mean, median, and mode of 3 sessions. The teaching sessions were 30–60 minutes. Two trained pediatric faculty raters were assigned to watch and assess each resident teaching video using Relate. Prior to assessing the videos, raters were trained on Relate instrument use. For the full study, in which trained raters assessed actual resident teaching videos, individual raters used Relate instrument to score 5 to 25 teaching videos each (median=11). The Relate instrument focused on 6 elements of resident teaching: learning environment, goals and objectives, promotion of understanding and retention, session management, and closure. These elements were further subdivided into 23 behaviors for which quality and frequency scales of “not at all,” “partially,” or “consistently” were included (Supplement 1). Data generated in this study is available at Dataset 1.

### Bias

To minimize bias, raters did not assess residents from their site and attested to not knowing any residents they assessed. Resident teaching videos and their assessments were de-identified.

### Study size

Sample size was not calculated a priori; we did a decision study as part of the generalizability study demonstrating how changing rater and observed teaching episode number affects overall Relate reliability.

### Statistical methods

 We collected validity evidence based on 4 sources as outlined by Messick’s unified framework for validity arguments: content, response process, internal structure, and relationship to other variables. Content as a source of validity evidence was explored previously; the remaining 3 sources are described here [7].

#### Response process

For response process, we analyzed rater accuracy and consistency (inter-rater reliability). Rater accuracy provides information on how closely raters align with expert-derived gold standard scores. Rater consistency provides a measure of rater agreement. Both statistics provide support on the quality of rater scores.

#### Rater accuracy

Prior to rater assessment of resident teaching, we conducted rater training for 6 novice raters. All raters were actively involved in resident education. We created 3 training videos of simulated resident-led case-based teaching. We developed expert-derived assessments of these videos by having the clinician instrument developers (A.S.F.V., E.Y.C., K.A.G.; the “expert consensus”) assess the videos and come to consensus on how to assess them using the assessment instrument. By expert consensus, we revised the behaviors on the instrument that could not be assessed objectively to improve objectivity. The final version of the instrument was named Relate. We then developed a guidebook for using Relate (Supplement 2) and a 3-hour workshop to train novice raters to assess resident teaching using Relate based on a Frame-of-Reference model as applied to medical education [8]. A team member (A.F.V.) trained novice raters at each site. There were 6 novice raters; all were pediatric faculty actively involved in resident education. We examined the change in novice raters’ accuracy on Relate pre- and post-training.

#### Rater consistency

For the full study, we used intraclass correlation (ICC) to measure interrater reliability to determine rater consistency. We determined the percentage of variance accounted for by the raters using a generalizability study (G-study).

#### Internal structure

We conducted a G-study to determine the reliability of scores on the rubric, accounting for different rating occasions, rater, and item effects. The G-study design was specified as occasion nested in learner crossed with raters and items, (occasion: learner)×(rater×item). We used the Φ-coefficient reliability to measure consistency across facets in the G-study design, as this was a criterion-referenced assessment. We conducted a decision study to determine reliability projections for varying the number of raters or teaching encounters that affect the instrument.

#### Relationship to other variables

To determine relationship to other variables, we compared the average total Relate score at the level of the learner to their placement on the Practice Based Learning and Improvement 8 (PBLI8) Pediatric Milestone: “develop the necessary skills to be an effective teacher” [9] using Pearson correlation. This milestone placement was done prior to Relate assessment and performed by the Clinical Competency Committee (CCC) for the Mass General for Children and Dartmouth residents as part of routine placement of residents on the milestones. Study authors (A.S.F.V., D.A.H., K.A.S., S.E.S.V.) were on the CCC at Mass General for Children. At Brown, the RAT rotation director (E.Y.C.) and chief residents placed the residents on the milestone at the end of their teaching rotation based on their rotation performance.

Data compilation and analyses were conducted using Stata ver. 16.0 (Stata Corp.). We used descriptive statistics to examine trends in assessment data. Kappa and weighted kappa (quadratically-weighted kappa) were used to examine rater accuracy and inter-rater reliability. Associations were examined using Pearson correlations.

## Results

### Participants

Sixteen residents (39.0% of all eligible residents) participated; 8 (44.4%) eligible from Mass General for Children, 5 (31.3%) from Brown, and 3 (42.9%) from Dartmouth. No demographic data was collected. To ensure that residents and programs are de-identified, programs are listed by letter (A, B, C) without identifying the number of resident subjects at each program.

### Main results

Table 1 provides the mean scores on each of the Relate sub-elements overall and by program. The highest scoring sub-element was: “created a respectful and open climate” (mean=1.61/2; standard deviation [SD], ±0.33). The lowest scoring element was: “explicitly encouraged further learning” (mean=0.14/2; SD, ±0.40).

#### Response process

Impact of rater training: Rater training improved agreement between raters and expert rating (exact agreement) by 36%. ICC estimates reflecting inter-rater reliability increased from 0.04 to 0.64 following rater training, demonstrating good inter-rater reliability [10] (Table 2).

Variability of raters during assessment: During full study rating, total variability due to raters and all associated rater interactions accounted for 2.1% of total variance, indicating high degree of consistency between assessors. Differences by rater for each learner accounted for 1.5% and by rater for item on Relate accounted 0.6% of total variance; variance due solely to rater severity effects was minimal. These findings reflect minor assessor-related effects on scores (Table 3).

#### Internal structure

**Fig. 1.**
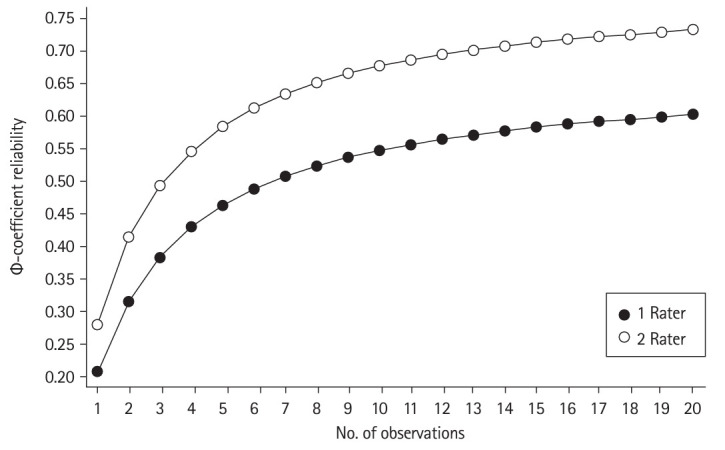
Decision study: projections in reliability given varying numbers of raters and occasions.

Reliability of assessment: Generalizability theory: Using variance components analysis based on generalizability theory, the overall assessment reliability (Φ-coefficient reliability) was 0.50, incorporating facets associated with occasion, rater, and item. Projections in reliability indicate improvement in reliability when 2 raters served as assessors (relative to a single assessor). Fig. 1 shows projections in reliability, indicating projected reliability of over 0.60 with 6 observations and 2 raters.

Variance components: Variability between items on the Relate instrument accounted for 26.2% of variance, which may reflect the difficulty in performing or in assessing the item. Variance due to learners accounted for 2.9% of overall variance, while occasion-related learner variance accounted for 3.9% of total variance. Further, 18.9% of total variance was accounted for by the combination of the learner and the rater or item (Table 3).

#### Relationship to other variables

The average total Relate score of each resident was associated with their placement on the PBLI8 Pediatric Milestone (r=0.34, P=0.019), which indicated a medium correlation.

## Discussion

### Key results

We collected multisite validity evidence supporting the response process, internal structure, and relations to other variables of Relate for faculty to assess resident-led large group teaching. Our data showed sufficient reliability of Relate to make decisions as part of workplace assessment [11]. Moreover, interrater reliability improved substantially. Scoring was sufficiently accurate and consistent to allow for confidence and precision of assessment data. Relate scores provide associations with Milestones that may yield future consequential impact. Therefore, the scores from Relate are reliable and demonstrate validity evidence for assessment of resident-led large group teaching. These data are helpful to residency programs and can also provide direct feedback to residents.

### Interpretation

Broadly, Relate demonstrates validity evidence, but it is also important to consider the individual components comprising the total variance of scores. It would be optimal if the learner facet led to a large difference in scores. The learner alone contributed to 2.9% to the overall variance, which is within range of prior observations and case-based assessment studies [12]. The lack of differentiation may be explained by our homogenous subject group of senior residents with likely similar competence levels. Relate assessments may better reflect the RAT curriculum or residency program training environment than individual teacher competence, and, as seen in Table 1, residents at the different site performed differently on different items of the Relate, likely due to differences in the RAT curricula at each site. We do not have enough teaching videos from each site to determine to what extent the curriculum or residency program itself contributed to assessment variability. The teaching occasion, which reflected the timing of each learner’s teaching experience relative to other teaching experiences, accounted for only 3.9% of the total variance, suggesting that additional teaching experiences did not lead to measurable improvement. RAT curricula may not meaningfully improve teaching or the learners may already be performing well as teachers leaving little room, or insufficient time between occasions, for measurable improvement. Other studies have found that competence changes slowly over time and that senior resident competence has limited variability and is more uniform than that of less experienced residents [13]. A much larger portion of the total variance (26.2%) came from the individual Relate item, reflecting that the Relate items were not all equally difficult (either to assess or to perform). Residents’ RAT curricula and teaching expectations may prioritize some, but not all, Relate items.

Relate scores correlated significantly with PBLI8 scores, suggesting that Relate assessments may be used to inform resident competency on teaching milestones. The moderate effect sizes indicate modest yet meaningful predictive associations. Given that rater training led to improved rater accuracy and agreement, programs must train faculty to use the tool to achieve good rater agreement. Frame-of-reference training was successful in improving interrater reliability for faculty using an instrument to assess faculty-led large group teaching [14].

### Comparison with previous studies

We were unable to identify prior instruments for faculty to assess either faculty-led or resident-led large group teaching with known validity evidence or that emphasized the interactive nature of teaching. Thus, we were not able to compare resident teaching performance on our instrument with any previously developed instruments. Only one study, which focused on prior Observed Structured Teaching Encounters (OSTE), conducted a generalizability study as part of their evidence of instrument validity and found the reliability to be 0.57 at one resident site and 0.62 at another [15] which is slightly higher than our findings, as would be expected when teaching was conducted in a controlled OSTE environment rather than a natural workplace. Relate contributes to the literature as an instrument with validity evidence for workplace assessment of resident-led large group teaching.

### Limitations

The resident participants were all in northeastern US pediatrics programs. There is no reason inherent to the instrument that would preclude the use of Relate in other specialties or programs. The study population included only senior residents who volunteered to participate which may have created a sampling bias. More score variance due to learners may have been identified if the study population included medical students, residents at different levels of training, and faculty. Residents had different numbers of teaching videos and those who had more videos contributed more data to the study population. This could have led to the decreased variance in the study population as well. We also had only 48 videos and residency programs had different numbers of videos. The residency programs were not uniform regarding the expectations of resident case-based teaching or the mentorship provided, which adds to generalizability. However, due to the small number of resident videos from each program and the over-representation of one program, we could not determine how these programmatic differences affected Relate scores. Further, the 3-hour rater training may be a barrier to Relate implementation. Having 6 observations and 2 raters, which was found to increase reliability of the instrument, may not be possible at all sites. In that case, Relate may still prove useful in guiding faculty members in giving feedback to residents on their teaching even without the interrater reliability that rater training provides.

### Generalizability

While additional studies would ensure generalizability, the Relate can be used broadly to assess teaching competence of residents which would fulfill this international requirement of program directors.

### Suggestions

There are several future directions. The interrater reliability of the instrument should be studied when raters are given the instrument and guidebook alone without the extensive training conducted in our study. An easier rater training program would increase feasibility of Relate use. Relate should be studied in other pediatric programs as well as in other specialties to determine its broader generalizability. It should also be studied with medical students, all levels of residents, and faculty as teachers. Finally, the impact of Relate on the resident’s teaching skill and ability to effectively transfer knowledge to learners would ideally be studied.

### Conclusion

Overall, we collected validity evidence using Messick’s framework for Relate as an instrument for faculty to assess resident-led large group teaching and found that it generates reliable scores with robust validity evidence.

## Figures and Tables

**Figure f2-jeehp-21-03:**
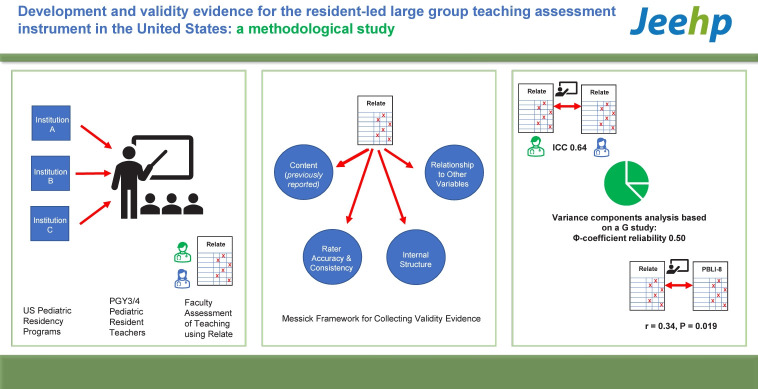


**Table 1.** Mean scores (0=“not at all,” 1=“partially” and 2=“consistently) for each Relate sub-element for all residents overall and by residency program; programs de-identified as program A, B, and C

Relate sub-element	Program A	Program B	Program C	Overall
Created a respectful and open climate	1.60±0.35	1.63±0.23	1.66±0.28	1.61±0.33
Clearly communicated the importance of the topic and encouraged participant engagement throughout the presentation	0.98±0.50	1.38±0.52	1.35±0.42	1.10±0.51
Set and communicated learner-centered, clear objectives appropriate for the time allotted	1.32±0.80	0.75±0.71	0.34±0.68	1.05±0.86
Demonstrated appropriate knowledge of the topic and used appropriate references	1.21±0.50	1.31±0.53	1.34±0.42	1.25±0.48
Tailored presentation level to participants’ understanding of the material	1.27±0.49	0.75±0.38	1.18±0.55	1.20±0.51
Explained concepts and interrelationships clearly	1.41±0.46	1.54±0.35	1.61±0.34	1.47±0.43
Used effective questioning and interactive techniques to promote learning and probed for supporting evidence or participants’ thought processes	1.09±0.51	0.54±0.47	1.42±0.50	1.12±0.55
Made efficient use of teaching time with appropriate pace and time spent on each objective and each component of the session	1.48±0.57	0.88±0.23	1.07±0.58	1.34±0.59
Content was logically organized with smooth transitions to assist comprehension and retention	1.70±0.50	0.88±0.35	1.14±0.47	1.50±0.56
Summarized key concepts and lessons learned	0.58±0.70	0.63±0.92	0.41±0.73	0.54±0.72
Explicitly encouraged further learning	0.14±0.43	0.13±0.35	0.14±0.35	0.14±0.40

Values are presented as mean±standard deviation. The number of resident subjects for each group is not included to preserve the confidentiality of the program identities. Relate, Resident-led Large Group Teaching Assessment Instrument.

**Table 2.** Comparison of interrater reliability to expert gold standard rating: pre- and post-training

Interrater reliability	Pre-training	Post-training
Exact agreement (%)	26	62
Kappa	–0.09	0.43
Weighted Kappa (intraclass correlations)	0.04	0.64

**Table 3.** Generalizability study^a)^: variance components

Effect (meaning associated with the effect)	df	VC	% VC
Learner (true differences between learners)	12	0.005	2.9
Occasion: learner (variation in learner performance by occasion)	32	0.006	3.9
Rater (variation in rater severity)	1	0.000	0
Item (variation in item difficulty)	22	0.042	26.2
Learner×rater (variation in learner performance by rater)	12	0.002	1.5
Learner×item (variation in learner performance by item)	264	0.018	11.0
(Occasion×rater): learner (variation in learner performance by rater and occasion)	32	0.009	5.6
(Occasion×item): learner (variation in learner performance by occasion and item)	704	0.021	13.3
Rater×item (variation in rater severity by item)	22	0.001	0.6
Learner×rater×item (variation in learner performance by rater and item)	264	0.006	3.9
Residual error (unexplained error)	704	0.050	31.2

df, degrees of freedom; VC, variance component.

a) Generalizability study using (occasion: learner)×(rater×item) design. Φ-coefficient reliability is 0.50.

## Footnotes

**Data availability**
Data files are available from Harvard Dataverse: https://doi.org/10.7910/DVN/2JF1E7 Dataset 1. Raw research data generated and analyzed in the current study.

## Supplementary materials

Supplementary files are available from Harvard Dataverse: https://doi.org/10.7910/DVN/2JF1E7

Supplement 1. Resident-led large group teaching assessment instrument (Relate).

Supplement 2. Relate rater guidebook.

Supplement 3. Audio recording of the abstract.
