# Supplementary material for: Development and validity evidence for the resident-led large group teaching assessment instrument in the United States: a methodological study
Source: J Educ Eval Health Prof. 2024 Feb 23;21:3. doi: 10.3352/jeehp.2024.21.3 (PMC10948941; doi:10.3352/jeehp.2024.21.3)
Supplement: Supplementary file 2 — Supplement 1. Resident-led large group teaching assessment instrument (Relate). [file jeehp-21-03-suppl1.pdf]

Supplement: Relate

Evaluator: Resident Codename/Video:

LEARNING ENVIRONMENT			
**A. Created a respectful and open climate**	Not at all	Partially	Consistently
1. Used respectful and inviting verbal and nonverbal language			
2. Promoted discussion of opinions, including divergent ones			
**B. Clearly communicated the importance of the topic and encouraged participant engagement throughout the presentation**	Not at all	Partially	Consistently
3. Explained importance of topic throughout presentation			
4. From the beginning, stimulated participants' interest in the topic (i.e. sharing practical advice, stories, challenges)			
5. Encouraged participants to examine their understanding and practice			
6. Conducted formal or informal assessment of participants' prior understanding of the topic throughout presentation			
**COMMENTS:**			
**GOALS AND OBJECTIVES**			
**C. Set and communicated learner-centered, clear objectives appropriate for the time allotted**	Not at all	Partially	Consistently
7. Objectives were actionable			
8. Session was organized around objectives.			
**COMMENTS:**			
**CONTENT OF TALK**			
**D. Demonstrated appropriate knowledge of the topic and used appropriate references**	Not at all	Partially	Consistently
9. Used literature appropriately			
10. Demonstrated appropriate knowledge of the topic			
**E. Tailored presentation level to participants' understanding of the material**	Not at all	Partially	Consistently
11. Targeted teaching points to multiple learner levels			
12. Related information to participants' prior knowledge or experience			
**COMMENTS:**			
**PROMOTION OF UNDERSTANDING AND RETENTION**			
**F. Explained concepts and interrelationships clearly**	Not at all	Partially	Consistently
13. Clearly explained concepts and reasoning			
14. Used clear, simple visual aids to demonstrate concepts			
15. Answered questions clearly and accurately			
**G. Used effective questioning and interactive techniques to promote learning and probed for supporting evidence or participants' thought processes**	Not at all	Partially	Consistently
16. Used questions to encourage sharing of knowledge or ideas			
17. Used questions to check for understanding of information taught.			
18. Included activities beyond Q&A that enriched participants' learning (i.e. pair or small group work, audience response system)			
**COMMENTS:**			
**SESSION MANAGEMENT**			
**H. Made efficient use of teaching time with appropriate pace and time spent on each objective and each component of the session**	Not at all	Partially	Consistently
19. Spent adequate time on each objective and each component of the session			
20. Facilitated discussion while minimizing digressions			
**I. Content was logically organized with smooth transitions to assist comprehension and retention**	Not at all	Partially	Consistently
21. Organized content logically to progressively build participants' understanding			
**COMMENTS:**			

**CLOSURE**			
**J. Summarized key concepts and lessons learned**	Not at all	Partially	Consistently
22. Summarized key learning points			
**K. Explicitly encouraged further learning**	Not at all	Partially	Consistently
23. Offered suggestions for further reading and/or learning activities			
**COMMENTS:**			

OVERALL TEACHING QUALITY:

novice
novice/advanced beginner
advanced beginner
advanced beginner/competent
competent
competent/proficient
proficient
proficient/expert
expert
