# Supplementary material for: Development and validity evidence for the resident-led large group teaching assessment instrument in the United States: a methodological study
Source: J Educ Eval Health Prof. 2024 Feb 23;21:3. doi: 10.3352/jeehp.2024.21.3 (PMC10948941; doi:10.3352/jeehp.2024.21.3)
Supplement: Supplementary file 3 — Supplement 2. Relate rater guidebook. [file jeehp-21-03-suppl2.pdf]

Rater Guide

- Anchors
  - Anchors (not at all/partially/consistently) refer to quantity (how much did they do it?) and quality (how well did they do it?)
  - If something was not applicable, select "not at all"

A. Created a respectful and open climate

1. Used respectful and inviting verbal and nonverbal language:
   - Not at all: statements, tone and/or body language were negative or dismissive to participants
   - Partially: statements, tone and/or body language were neutral, neither positive or negative, neither encouraging nor dismissive, or balance of positive and negative comments/tone such that the positive and negative evens out
   - Consistently: statements, tone and/or body language were positive and encouraging to participants

2. Promoted discussion of opinions, including divergent ones:
   - Not at all: not at all open to discussion of other opinions
   - Partially: took into account some opinions of audience or topic did not lend itself to people having opinions
   - Consistently: actively sought/brought up a variety of views

B. Clearly communicated the importance of learning about the topic and encouraged participant engagement throughout the presentation

3. Explained importance of topic throughout the presentation
   - Not at all: did not explain the value or importance at all
   - Partially: explained value or importance at some point in the presentation
   - Consistently: emphasized the value or importance of the topic at least at the beginning and the end of the presentation

4. From the beginning, stimulated participants' interest in the topic (i.e. sharing practical advice, stories, challenges)
   - Not at all: not stimulating/motivating at all
   - Partially: stimulating/motivating during parts of the presentation, but not from the beginning or not consistently throughout
   - Consistently: from the beginning, got people hooked, offered personal challenges, asked a question to think about, etc., that makes the topic of the presentation interesting

5. Encouraged participants to examine their understanding and practice
   - Not at all: participants not asked to examine understanding or practice
   - Partially: participants asked to examine understanding OR practice
   - Consistently: participants asked to examine understanding AND practice – must include both WHAT they would do and WHY

6. Conducted formal or informal assessment of participants' prior understanding of topic throughout the presentation
   - Not at all: no attempt to understand prior understanding of topic
   - Partially: tried to assess understanding at some point in the presentation
   - Consistently: tried to assess what the participants already know several times – must be for WHOLE audience, not just select levels of learners

C. Set and communicated learner-centered, clear objectives appropriate for the time allotted

7. Objectives were actionable
   - Not at all: no objectives or objectives were implicit or they were not actionable

- Partially: objectives were presented and some were actionable
- Consistently: objectives were presented clearly and all were actionable

8. Session was organized around objectives
- Not at all: the presentation did not appear to follow or cover the objectives or there were no objectives
- Partially: the presentation generally followed the objectives, but perhaps not in an organized fashion or spent a lot of time on an aspect that was a tangent or was not one of the objectives
- Consistently: the presentation clearly moved from one objective to the next in an organized fashion

D. Demonstrated appropriate knowledge of the topic and used appropriate references

9. Used literature appropriately
- Not at all: did not reference any literature for the presentation or made a vague reference to having done some reading
- Partially: clearly used literature and explained where the literature came from, though used a secondary source only such as UpToDate alone; if using homegrown tools, did not explain source
- Consistently: identified literature used and used the appropriate references (guidelines, some primary literature); if used homegrown tools, explained that these were based in the literature

10. Demonstrated appropriate knowledge of the topic
- Not at all: did not understand the material covered or gave incorrect information
- Partially: had a basic understanding of the material covered but had some significant holes in knowledge. Of note, the resident is NOT supposed to be the expert on the topic, so it is expected that the resident will have some holes in knowledge
- Consistently: had a good understanding of the material and when there were aspects that were unknown, was honest about it, sought help in finding answers from the participants

E. Tailored presentation level to participants' understanding of the material

11. Targeted teaching points to multiple learner levels
- Not at all: level of discussion did not vary throughout, only targeted one level of learner
- Partially: some variation in level taught addressed, but did not target all levels (med students to faculty)
- Consistently: targeted different points to each level of learner ensuring that there was something of interest for each level of learner

12. Related information to participants' prior knowledge or experience
- Not at all: talk did not acknowledge participants' experience or knowledge (even if solicited), just moved on to topic that may be unrelated to this prior knowledge/experience
- Partially: acknowledged experience/knowledge and info presented next related to this prior knowledge/experience
- Consistently: incorporated the prior experience/knowledge explicitly into presentation of new information, may even reference that prior experience/knowledge at different point in talk

F. Explained concepts and interrelationships clearly

13. Clearly explained concepts and reasoning
- Not at all: vague or confusing presentation of information
- Partially: described some concepts clearly but not all
- Consistently: uses language that is understandable to audience, described reasoning with an explicit organizational framework

14. Used clear, simple visual aids to demonstrate concepts
- Not at all: no visual aids

- Partially: visual aids too complex or did not clearly demonstrate concepts – visual aid can be handout, doesn't have to be on the screen/board
- Consistently: visual aids clear, simple, and demonstrate concepts well – visual aid can be handout, doesn't have to be on the screen/board

15. Answered questions clearly and accurately
- Not at all: no questions, or responses that did not answer the question asked
- Partially: vague answers that only partially responded to the question; repeated prior information presented without further explanation; may have answered some questions well but not all
- Consistently: used clear and specific language; expanded on prior information or examples; checked for understanding of the person who asked the question (by looking for non-verbal language such as head nodding or asking directly, "does that answer your question")

G. Used effective questioning to promote learning and probed for supporting evidence or participants thought processes

16. Used questions to encourage sharing of knowledge or ideas
- Not at all: questions were not used to encourage participants to share knowledge
- Partially: used some questions to encourage sharing, but could have used more and/or questions could have been structured better
- Consistently: used open-ended questions for a specific purpose (generating ideas, understanding audience perspective, etc.) and more specific directive or guiding questions for appropriate reasons (such as guiding the discussion or helping to draw out points from the audience in follow-up to an open-ended question); allowed enough time for participants to respond

17. Used questions to check for understanding of information taught
- Not at all: questions were not used to check for understanding
- Partially: scattered questions that may test recall of some information taught, questions did not test for understanding
- Consistently: audience asked to apply main concepts taught (i.e.: quiz or cases at end of session or applying knowledge generated in one part of talk to cases in another)

18. Included activities beyond Q&A that enriched participants' learning (i.e. pair or small group work, audience response system)
- Not at all: no integration of activities beyond questions directed at participants
- Partially: incorporated activities beyond participant-directed questions (such as small group, think-pair-share, audience response system, etc.); however, activity did not appear to enhance learning
- Consistently: incorporated activities beyond participant-directed questions (such as small group, think-pair-share, audience response system, etc.) which effectively enhanced learning

H. Made efficient use of teaching with appropriate pace and time spent on each objective and each component of the session

19. Spent adequate time on each objective and each component of the session
- Not at all: insufficient time allotted to cover each objective or component of talk; resident ran out of time on the majority of objectives or components of session
- Partially: resident ran out of time on a few objectives or components but was able to cover most adequately
- Consistently: resident spent adequate time on each objective and component of talk

20. Facilitated discussion while minimizing digressions

- Not at all: did not attempt to facilitate discussion among participants but rather lectured or directed questions at individuals without inviting any cross-talk or discussion on a topic OR participants completely directed discussion which digressed from objectives
- Partially: attempted to facilitate discussion in which an exchange of ideas between participants occurred and either allowed for some tangents which took away from discussion or did not guide the discussion enough to cover objectives well
- Consistently: participants were truly engaged in a discussion beyond being asked to answer questions in which a true exchange of ideas took place. Guided the course of the discussion to meet objectives and did not let tangents detract from the discussion

I. Content was logically organized with smooth transitions to assist comprehension and retention

21. Organized content logically to progressively build participants' understanding
    - Not at all: content was disorganized and did not follow a logical order or thought process; jumped from one concept to another
    - Partially: content was presented in a standard textbook order, such as epidemiology, clinical presentation, diagnosis, and management but did not connect back to case or participants' thought process
    - Consistently: content was presented in an order that follows participants' thought process and progressively built on information presented

J. Summarized key concepts and lessons learned

22. Summarized key learning points
    - Not at all: no summary of key learning points provided
    - Partially: emphasized few key learning points during talk but did not summarize at end for participants
    - Consistently: provided key learning points throughout talk and summarized at end for participants

K. Explicitly encouraged further learning

23. Offered suggestions for further reading and/or learning activities
    - Not at all: did not provide any suggestions for further reading or learning activities
    - Partially: provided some general non-specific ideas for extension of learning
    - Consistently: provided suggestions for further reading or learning opportunities

- Overall teaching quality: purposefully not a summation or average of the behavioral checklist – overall perception
